# Real-world experience with 240 mg of galcanezumab for the preventive treatment of cluster headache

**DOI:** 10.1186/s10194-022-01505-w

**Published:** 2022-10-08

**Authors:** Heejung Mo, Byung-Kun Kim, Heui-Soo Moon, Soo-Jin Cho

**Affiliations:** 1 Department of Neurology, Dongtan Sacred Heart Hospital, Hallym University College of Medicine, Keun Jae Bong-gil 7, Hwaseong, Gyeonggi-do 18450 South Korea; 2 Department of Neurology, Nowon Eulji Medical Center, Eulji University School of Medicine, Daejeon, South Korea; 3 Department of Neurology, Kangbuk Samsung Hospital, Sungkyunkwan University School of Medicine, Seoul, South Korea

**Keywords:** Cluster headache, Galcanezumab, Migraine, Preventive treatment

## Abstract

**Background:**

Galcanezumab of 300 mg monthly is the FDA approved preventive medication for cluster headache (CH) during the cluster period. Compared to the 120 mg galcanezumab syringe for the treatment of migraines, the 100 mg syringe for CH has globally not been as widely available. The aim of our study was to investigate the preventive efficacy and tolerability of two 120 mg galcanezumab doses for episodic CH in clinical practices.

**Methods:**

We evaluated patients with CH who received at least 1 dose of 240 mg (2 prefilled syringe of 120 mg) of galcanezumab in the 3 university hospitals from February 2020 to September 2021. In the patients with episodic CH, the efficacy and safety data of galcanezumab were analyzed regarding to the presence of the conventional preventive therapy at the timing of therapy of galcanezumab. The data of other subtypes of CH were separately described.

**Results:**

In 47 patients with episodic CH, galcanezumab was started median 18 days after the onset of current bout (range 1–62 days) and 4 patients (10.8%) received second dose of galcanezumab. The median time to the first occurrence of 100% reduction from baseline in CH attacks per week after galcanezumab therapy was 17 days (25% to 75% quartile range: 5.0 ~ 29.5) in all patients with episodic CH, 15.5 days (3.8 ~ 22.1) in 36 patients with galcanezumab therapy add-on conventional preventive therapy, 21.0 days (12.0 ~ 31.5) in 11 patients started galcanezumab as initial preventive therapy. Among 33 patients with headache diary, the proportion of patients with 50% or more reduction in weekly CH attacks at week 3 from baseline were 78.8%. There was no significant difference in the proportion of patients with a reduction of at least 50% in weekly frequency of CH attacks at week 3 between 24 patients received galcanezumab therapy add-on conventional preventive therapy and 9 patient who received initial galcanezumab therapy. (83.3%, vs 66.7%, *p* = 0.36). There were no significant differences in proportion of “very much better or “much better” between 36 patients received galcanezumab therapy add-on conventional preventive therapy and 11 patient who received initial GT (86.1%, vs 63.6%, *p* = 0.18).

**Conclusion:**

One 240 mg dose of galcanezumab with/without conventional therapy for the prevention of CH is considered effective and safe in clinical practices, as seen in the clinical trial of galcanezumab.

## Introduction

Cluster headache (CH) is a disabling primary headache disorder characterized by clustering of severe headache attacks lasting between 15 and 180 minutes. Prophylactic therapy is recommended from the onset of the cluster period or bout [1, 2]. The burden of cluster headaches are so severe that it significantly impairs the occupational life and work efficacy of those impacted [3]. It is also known to be associated with increased emotional stress and suicidal idea [4].

There are various known therapeutic approaches to treat CH: traditional preventive therapy such as verapamil or lithium, and transitional therapy such as sub-occipital steroid injection or short-term steroid therapy. Several possible effective therapies are recommended with level C evidence: valproic acid, topiramate, melatonin, baclofen, frovatriptan, and warfarin (only for the patients with chronic cluster headache) [1, 2]. However, only one-third of the patients with episodic CH and half of the patients with chronic CH opt for prophylactic treatment [5]. This low adherence of prophylaxis may be partly due to the adverse events (AE) associated with the medication and patients being uninformed about the importance of prevention.

Galcanezumab, a monoclonal antibody targeting calcitonin gene-related peptide, is the first and the only FDA approved preventive medication for both episodic CH and migraine [6–8]. The approved dosage of galcanezumab for CH is 300 mg monthly during the cluster period and that for migraine is 120 mg monthly after 240 mg loading dose. More than 70% of CH patients, on a 300 mg galcanezumab dose, were reported a reduction of at least 50% in the weekly frequency of cluster headache attacks at week 3 in that trial with a dose of 300 mg of galcanezumab [6]. Galcanezumab may have some merits of rapid efficacy and low AE [9].

Compared to the approval and availability of the 120 mg galcanezumab syringe for the treatment of migraines, the 100 mg syringe of galcanezumab for CH has been unavailable in several countries including Korea. The 1-year prevalence of CH was estimated to be 53–119 per 100,000, consequently its rarity may halt the proper induction of its efficacy as a proven treatment. A retrospective analysis of off-label treatment attempts showed that a 240 mg dose of galcanezumab or a 70–140 mg dose of erenumab for chronic CH had comparable efficacy [9]. Approximately 15% of CH patients also reported having comorbid migraine, for which clinician can accordingly offer a loading dose of 240 mg galcanezumab [10].

We investigated the preventive efficacy and tolerability of two 120 mg galcanezumab doses for episodic cluster headaches in clinical practices [9, 11, 12].

## Materials and methods

### Study design and patients

**Fig. 1:**
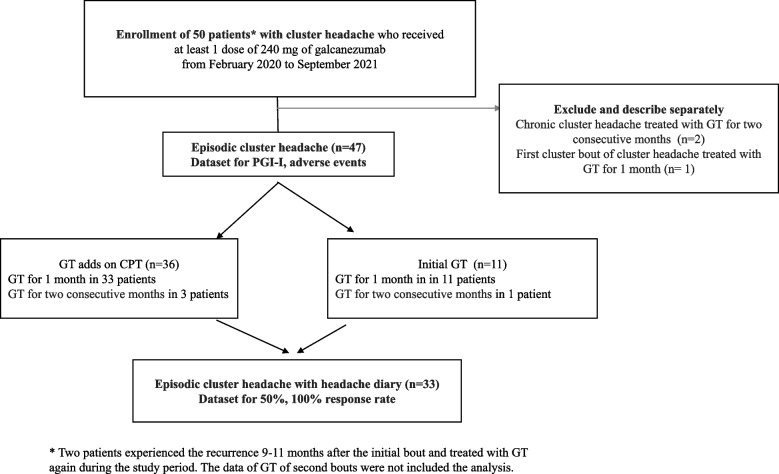
Selection of patients with cluster headache. GT galcanezumab therapy, CPT conventional preventive therapy, PGI-I, Patient global impression of improvement

In this multi-centered observational study, we collected the data of patients with CH who received at least one 240 mg galcanezumab dose (2 prefilled syringe of 120 mg) at the 3 university hospitals, from February 2020 to September 2021. The eligible participants were 18 to 60 years of age, and the diagnosis of episodic CH (ECH) was according to the diagnostic criteria of the International Classification of Headache Disorders (ICHD), 3rd edition [13]. Investigators carefully evaluated the patients and made the CH diagnosis based on the patient’s history and clinical presentation using the third edition of the ICHD. We excluded the data of galcanezumab therapy (GT) for second cluster bout in 2 patients in this analysis due to duplication of the same patients (Fig. 1).

The study protocols of prospective and retrospective registry were approved by the institutional review board (IRB) at each hospital (EMCS 2021–10–032-001). For the patients who received galcanezumab for prevention of cluster headache before the IRB approval, IRB allowed the process of written informed consent to be waived due to retrospective data collection and fully anonymity. After IRB approval, all patients were given full explanation of the study purposes and provided written informed consent before their voluntary participation. This study was conducted in accordance with the principles of the Declaration of Helsinki.

### Treatment

The decision to use GT for prevention of CH was made of the patients own volition after the investigator’s recommendation, as this specific course of treatment for CH is not covered by the Korean national insurance. Only a 120 mg syringe of galcanezumab was available in Korea, 1 dose of 240 mg (2 prefilled syringe of 120 mg) of galcanezumab was given to patients with CH. There was no restriction regarding the usage or dosage of any other abortive and preventive medication besides GT. After 1 month of initial injections, the second dose of galcanezumab was recommended, but the treatment decision was made based on the status of remission and patient’s preference.

### Data collection

The following demographic data was extracted from the patients’ electronic medical records: onset and end date of cluster bout and feature of CH, acute and preventive treatment, and history of comorbid migraine. Patients from the prospective registry were asked to keep a headache diary and record the frequency of CH attacks, the number of days with acute medication, and the pain severity. Patient global impression of improvement (PGI-I) and adverse drug responses were assessed 4 weeks after the last dose of galcanezumab. The following information about patients from the retrospective registry were obtained by medical records or telephonic interviews: information about the last day of cluster bout, PGI-I, and adverse drug responses.

### Efficacy and safety assessments

The efficacy and safety data of galcanezumab were analyzed in patients with ECH according to the timing and the approach of GT: whether GT was added onto the conventional preventive therapy (CPT), or whether GT was started as the initial preventive therapy. Median time to first occurrence of 100% baseline reduction (remission) in CH attacks after the first GT was assessed by timing of GT and the week after GT. PGI-I and adverse response were assessed by the timing of GT.

Fifty % reduction from baseline to 3-week in CH attacks per week and the days with acute medications per week were assessed in patients with headache diary who enrolled for prospective registry.

The data of other subtypes of cluster headache, such as chronic cluster headache, probable cluster headache, or the first episode of cluster bout were separately described.

Safety assessment data were collected from the patients’ EMR, self-reported headache diary, or telephone interviews.

### Statistical analyses

Patient baseline characteristics and clinical features were the two-sample *t*-test and Mann-Whitney U-test were used to compare the mean values according to whether or not each variable conformed to a normal distribution. The normality of data distribution was evaluated by the Shapiro-Wilks test. The chi-square test or Fisher’s exact test was used to compare categorical variables. All tests were two-tailed, and a *p*-value < 0.05 was considered to represent statistical significance. All analyses were performed using R for Windows (ver. 4.1.2; R Foundation for Statistical Computing, Vienna, Austria) and RStudio (ver. 2022.02.0 + 443; RStudio, Boston, MA, USA).

## Results

### Selection of enrolled patients, baseline characteristics, and prevention with Galcanezumab therapy

Fifty patients with CH who received at least 1 dose of 240 mg (2 prefilled syringe of 120 mg) of galcanezumab were enrolled during our study period. Two patients with chronic CH and one patient during his first cluster episode were excluded from the analysis for the GT efficacy in episodic cluster headache (ECH) (Fig. 1).

The mean age of the 47 patients with ECH was 40.4 (range 25–61) years and they had experienced 2–28 bouts before the current bout. The 47 patients were comprised of 39 males (83.0%) and 8 females. Thirteen patients (27.7%) had a previous history of migraines.

Regarding the timing of GT for current cluster bout: 36 patients added the GT on their CPT and 11 patients started GT as their initial preventive therapy. During total period of preventive therapy, 2 patients received three preventive drugs (verapamil, lithium, and other drug such as candesartan), 21 patients received two preventive drugs (verapamil and topiramate 15, verapamil and lithium 4, topiramate and other medications 1, verapamil and other drug 1), 12 patients received one preventive drug (verapamil 8, lithium 3, other drug 1), and 1 patient received only transitional therapy. In the initial GT group, mean onset age of CH was about 5 years younger and disease duration of cluster headache was somewhat longer than GT with CPT group. However, there was no significant difference of baseline characteristics between the two groups including psychiatric comorbidities or suicidal idea (Table 1).

**Table 1:** Baseline characteristics of the patients with episodic cluster headache according to the timing of 240 mg of galcanezumab therapy (GT)

	GT add-on CPT (*n* = 36)	Initial GT (*n* = 11)^a^	*P*-value
Age, years	40.1 ± 8.7	41.5 ± 9.4	0.68
Male sex, n (%)	29 (80.6)	10 (90.9)	0.73
Onset age, years	29.5 (22.0, 35.3)	24 (20.0, 29.5)	0.20
Duration of CH disease, years	8.5 (5.0, 12.5)	10 (8.0, 21.5)	0.07
Average duration of cluster period, weeks	6 (5.0, 8.0)	8 (4.5, 10.0)	0.67
time to GT from the onset of cluster bout, days	19 (13.2, 28.2)	9.0 (8.5, 23.5)	0.23
BMI, kg/m^2^	24.3 ± 4.3	23.5 ± 1.9	0.41
Ever-smoker, n (%)	22 (61.1)	7 (63.6)	1.00
Current alcohol drinking, n (%)	19 (52.7)	7 (63.9)	1.00
Comorbid migraine, n (%)	11 (30.4)	2 (18.2)	0.68
PHQ-9 score*	7.9 ± 6.6	9.9 ± 7.3	0.50
GAD-7 score*	8.5 ± 5.5	10.6 ± 7.2	0.42
EQ-5d scores*	0.91 (0.86, 1.00)	0.84 (0.79, 0.94)	0.46
Passive suicidal idea*	70.5%	87.5%	0.62
Abortive treatment			
Oxygen, n (%)	10 (27.8)	3 (27.2)	1.00
Triptan, n (%)	29 (80.6)	4 (36.4)	0.26
CPT			
Verapamil, n (%)	27 (75.0)	–	
Lithium, n (%)	6 (16.7)	–	
Prednisolone, n (%)	26 (72.2)	–	
Occipital nerve block, n (%)	23 (63.9)	–	
Topiramate, n (%)	14 (38.9)	–	

Age and BMI are presented as mean (standard deviation). The remaining data are presented as median (quartile) according to normality of variable

*GT* galcanezumab therapy, *CPT* conventional preventive therapy, *CH* cluster headache, *BMI* body mass index

*Data about psychiatric comorbidities and suicidal idea were available among 26 patients (GT add-on CPT 17, Initial GT 8). No patient attempted suicide

^a^Five patients added other conventional preventive therapies after the start of GT

Galcanezumab of 240 mg was injected an average of 18 days after the onset of current bout (range 1–62 days). Among 12 patients who had ongoing attacks 1 month after GT, 8 patients were initially included GT add-on CPT and 4 were included in initial GT group. Four patients, 3 patients in GT add-on CPT and 1 patient in initial GT group, received the second galcanezumab dose of 120 or 240 mg an average of 31 days after initial GT.

### Occurrence of 100% and 50% reduction in CH attacks and days with acute medications after 240 mg of GT among ECH

Median time to the first occurrence of 100% reduction from baseline in CH attacks per week after the first GT was 17 days (25% to 75% quartile range: 5.0 ~ 29.5) in 47 patients with ECH. 100% reduction in CH attacks per week were achieved within 1 week in 13 patients (27.7%), within 2 weeks in additional 10 patients (21.3%), within 3 weeks in 6 more patients (12.8%). Finally, 35 patients got remission 1 month after GT.

Regarding the timing of GT, median time to first occurrence of 100% reduction from baseline in CH attacks per week was 15.5 days (3.8 ~ 22.1) in 36 patients with GT add-on CPT, 21.0 days (12.0 ~ 31.5) in 11 patients with initial GT, and 12.5 days (12.0 ~ 19.8) in 6 patients with GT as sole prevention. No recurrence was observed within 3 months after the occurrence of 100% reduction from baseline in CH attacks.

The efficacy of GT was analyzed about a reduction of at least 50% in weekly frequency of CH attacks and the days with acute medications per week at week 3 from baseline in 33 patients with headache diary data. The mean numbers of CH attacks were decreased from 8.6 attacks (SD 4.8) in baseline to 1.8 attacks (SD 2.4) in week 3. The median number of weekly CH attacks and the median days with acute medication per week significantly decreased after GT (Table 2). At week 3, the proportion of patients with a 50% or more reduction in weekly CH attacks was 78.8% and the proportion with a 50% or more reduction in days with acute medication per week was 79.3%. There was no significant difference in the proportion of patients with a reduction of at least 50% in weekly frequency of CH attacks at week 3 between 24 patients received GT add-on CPT and 9 patient who received initial GT. (83.3%, vs 66.7%, *p* = 0.36).

**Table 2:** Efficacy of 240 mg of galcanezumab therapy at week 3 compared to baseline in patients with episodic cluster headache based on the headache diary (*n* = 33)

	Baseline	Week 1	Week 2	Week 3	Week 4	Patients with a reduction of at least 50%, n (%)	Patients with a 100% reduction, n (%)
Number of attacks per week	7.0 (6.0, 10.0)	4.0 (1.0, 6.0)	4.0 (0, 6.0)	0 (0, 4.0) ^b^	0.0 (0, 1.0)	26 (78.8)	18 (54.5)
Days with acute medications per week^a^	7.0 (3.0, 7.0)	1.5 (0.0, 4.0)	1 (0, 3.0)	0 (0, 1.3) ^b^	0 (0, 0)	23 (79.3)	18 (62.1)
Pain intensity during attacks [0–10]	8.0 (7.0, 9.0)	6.0 (4.8, 7.3)	4.5 (0, 5.3)	0 (0, 5.0) ^b^	0 (0, 1.8)	NA	NA

Data is presented as median (quartile) according to normality of variable

^a^4 patients did not take any oral abortive medications during baseline

^b^*P*-value < 0.001, comparison from baseline to week 3

Among 12 patients who had ongoing attacks 1 month after GT, 4 patients who received the second GT had finished their CH bout 52–66 days after the first dose of GT (19–35 days after the second dose of GT) and 8 patients who did not receive the second GT finished their CH bout 31–99 days after the first dose of GT.

### Patient global impression of improvement and adverse response after galcanezumab therapy

**Fig. 2:**
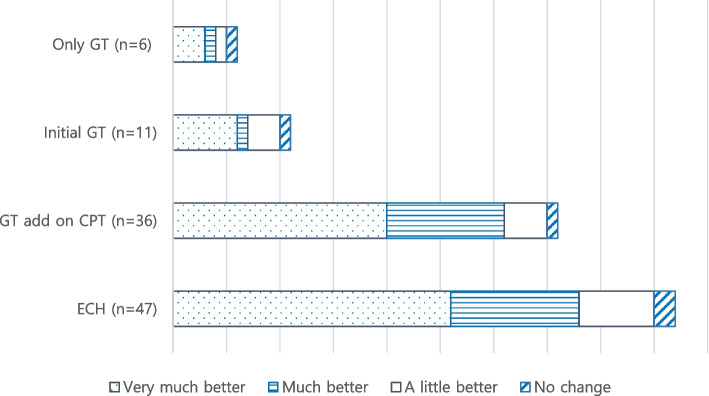
Patient global impression of improvement of galcanezumab therapy. GT galcanezumab therapy. CPT conventional preventive therapy, ECH episodic cluster headache

Among 47 patients with ECH, PGI-I were reported as feeling “very much better” in 26 patients, “much better” in 12 patients, “a little better” in 7 patients, and “no change” in 2 patients. No patients reported feeling of any worse. The proportion of “very much better” or “much better” was 80.9% in 47 patients with ECH. There were no significant differences in proportion of “very much better or “much better” between 36 patients received GT add-on CPT and 11 patient who received initial GT (86.1%, vs 63.6%, *p* = 0.18). There were no definite differences in the proportion of “very much better” or “much better” according to presence of transitional therapy such as sub-occipital steroid injection or short-term steroid therapy. (86.4% vs. 60%, *p* = 0.08, Fig. 2).

No serious AE occurred during the study period. More than half of the patients in both groups did not report any AE during GT (61.1% vs 72.7%, *p* = 0.72, Table 3). There were no differences in the frequency of AE according to the timing of GT.

**Table 3:** Adverse events of 240 mg of galcanezumab therapy in patients with episodic cluster headache

	GT add-on CPT (*n* = 36)	Initial GT (*n* = 11)^a^	*p*-value
None	22 (61.1)	8 (72.7)	0.72
Constipation	11 (30.6)	2 (18.2)	0.70
Hiccup	1 (2.7)	0	1.00
Myalgia	0	1 (9.1)	1.00
Neck pain	1 (2.7)	0	1.00
Injection-site swelling	1 (2.7)	0	1.00
Nocturia	1 (2.7)	0	1.00

*GT* galcanezumab therapy, *CPT* conventional preventive therapy

^a^Five patients added other conventional preventive therapies after the start of GT

### Experience of galcanezumab therapy in 2 patients with chronic CH and 1 patient with first cluster bout

A 24-year-old male patient with primary chronic CH, enrolled 6 years after the onset of cluster bout and remitted after 2 months of consecutive GT. A 19-year-old male with secondary chronic CH enrolled 7 months after the onset of the cluster period and remitted 3 months after consecutive GT. A 29-year-old male in the first episode of cluster bout enrolled 2 months after the onset of cluster headache and remitted 24 days after GT. The patients had several conventional preventive therapies added onto the GT and none of them had a history of migraine. PGI-I were reported as feeling “very much better” in a patient primary secondary chronic CH and “much better” in a patient secondary chronic CH and a patient with the first episode of cluster bout. Only one patient with primary chronic CH reported mild constipation after GT.

## Discussion

The main findings of our real-world study of GT over 20-months for the prevention of CH, were follows: (1) one dose of 240 mg of GT with/without conventional therapy for prevention of CH is effective in this study. Median time to remission after the first GT was 17 days and the proportion of patients with 50% reduction at week 3 from baseline about the numbers of CH attacks per week was 78.8%: (2) Patient with ECH received GT about 2 weeks after the onset of cluster bout and 91.5% of patients with ECH received GT just once in clinical practice; (3) In patients with relatively low frequency of CH attacks, as observed in Asians, the efficacy of GT with/without CPT was comparable to Western data; (4) GT was safe and well tolerable with/without CPT in patients with CH; (5) If other treatments are ineffective during several months of cluster bout, adding GT can be a good option to get much better improvement or obtain remission in patient with CH, even in patients with chronic CH or the first cluster bout.

This observational study was similar in the following ways to the historical randomized clinical trial (RCT) published in 2019 [6]: in mean age, sex ratio, and numbers of the participants who received galcanezumab. The following differences were noted between the two studies: proportions of smoking exposure (62% in this study, 79% in the RCT), the number of CH attacks per week in baseline (7.9 in those with diary data vs 17.8 in the RCT), and combination of other preventives (not allowed in the RCT) were different between two studies. Regarding the efficacy of GT of the 33 patients with headache diary in this study, the percentage of patients with at least 50% reduction in headache frequency at week 3 was 78.8% (71% in the RCT) and mean reduction of in the weekly CH frequency at week 3 was 6.8 attacks (8.7 attacks in the RCT). Our results supported that the treatment effect in observational studies was reported as similar to those obtained in RCT [14]. The real-world situation is not similar to the RCT conditions, but similar efficacy may be mixed effect of variability of status of patients and combined treatment in actual practices.

In this study, 91.5% of ECH received only one GT and 74.5% of ECH went into remission within 1 month after GT. We cannot rule out the influence of delayed start of GT and relatively shorter cluster bout on this one-shot GT efficacy. Although CH patients in Asia may have low proportion of smoking exposure, a lower attack frequency, and shorter bout duration [15, 16] compared to European and American populations, the efficacy of GT may be similar worldwide. The higher percentage of “very much better” or “much better” by PGI-I after GT also supported this conclusion. Considering only 1 patient was included in the RCT, this study can give practical information about GT for Asian CH patients.

The best time for GT in ECH is uncertain. Many patients are unable to come to the clinic from the onset of their cluster bout. The average duration from onset before GT intervention was about 2 weeks. Some CH patients were able to predict the upcoming bout based on early symptoms prior to the active bout. CH attacks may be less severe, less frequent, or shorter or longer duration especially around beginning and end of cluster bout [13, 17]. Whether early GT intervention can shorten the duration of cluster bout remains elusive and requires further evaluation.

Reports of AEs of GT were variable, none were serious, and most were well tolerable with/without CPT. The risk of AEs was reported as relatively high after verapamil or lithium or galcanezumab, but the burden of AEs is reasonable in the patients with GT and conventional preventive therapies. Prescription medication for the prevention of constipation, as is the usual practice for CH patients, may influence this result.

Our study had several limitations. First, the observed efficacy of GT can be an effect of spontaneous remission. Average duration of previous CH bouts was 6–8 week and the interval between onset of CH bout was 18 days, so it seems some patients’ remission might be due to the natural course of their bout. The actual efficacy of GT may be re-evaluated as an initial treatment for the next CH bout. Although placebo group was lack, PGI-I of the patients with long duration of cluster disease may support the efficacy of GT. Second, it is impossible to separate the efficacy of GT from that of CPT. Discontinuation of other preventive therapies after GT or withhold before GT can be also dilemma to both physicians and patients. Third, sample size of this study was too small to assess the efficacy of GT with various combination of conventional preventives, different starting date of GT, and personal diversity of cluster period. The role of transitional therapy after GT is reasonable to evaluate in a larger number of patients. Finally, we cannot avoid selection bias from university hospital setting with special interest in CH and data with/without headache dairy. There are no differences in age, sex, and life-time duration of cluster disease between those with and without headache dairy in this study. In addition, age, sex, and percentage of smoking exposure was similar to those in the patients of Korean Cluster Headache Registry. However, these results could only reflect a group of referral or more severely affected CH patients and may not well represent the real-world situations of the total CH populations.

## Conclusions

GT may be effective and safe in the treatment of ECH with or without CPT, even 2 weeks after cluster bout onset.

## Data Availability

The data used in the present study are available from the corresponding author on reasonable request.

## Abbreviations

AE: Adverse events
CH: Cluster headache
CPT: Conventional preventive therapy
ECH: Episodic cluster headaches
GT: Galcanezumab therapy
ICHD: the international classification of headache disorders
IRB: Institutional review board
KCHR: Korean cluster headache registry
PGI-I: Patient global impression of improvement
RCT: Randomized clinical trial
